# Bilateral delayed endolymphatic hydrops evaluated by bilateral intratympanic injection of gadodiamide with 3T-MRI

**DOI:** 10.1371/journal.pone.0206891

**Published:** 2018-12-05

**Authors:** Yoh-ichiro Iwasa, Keita Tsukada, Masafumi Kobayashi, Tomohiro Kitano, Kentaro Mori, Hidekane Yoshimura, Hisakuni Fukuoka, Shin-ichi Usami

**Affiliations:** Department of Otorhinolaryngology, Shinshu University School of Medicine, Matsumoto, Japan; University of Miami School of Medicine, UNITED STATES

## Abstract

The purpose of this study was to assess the diagnostic performance of 3T MRI after intratympanic injection of gadodiamide for delayed endolymphatic hydrops (DEH), and assess the relationship between endolymphatic hydrops (ELH) and vestibular function in patients diagnosed with DEH and confirmed by 3T MRI. Nineteen patients clinically diagnosed with DEH (11 ipsilateral DEH, 8 contralateral DEH) participated in this study. Diluted gadodiamide was administered to the bilateral tympanic cavity by injection through the tympanic membrane. At 24 hours post-injection, the ELH was evaluated by MRI. Patient vestibular functions were evaluated by caloric testing and cVEMP. ELH was observed in all patients (19/19: positive rate 100%). The distribution patterns of ELH varied between the cochlear or vestibular region. Vestibular ELH was observed in the affected ear in all ipsilateral DEH patients. In the contralateral DEH patients, however, there were individual differences in the distribution patterns of ELH. Six patients (1 ipsilateral DEH, 5 contralateral DEH) had bilateral ELH. No obvious relationships were observed between ELH and vestibular function. ELH distribution was complicated, particularly in the contralateral DEH cases. It was difficult to identify the existence of ELH by vestibular functional testing alone; therefore, 3T MRI is thought to be useful for identifying the affected ear. A significant number of cases had “bilateral” DEH, particularly among the contralateral DEH cases, indicating that we should pay careful attention to this pathology when treating DEH.

## Introduction

Delayed endolymphatic hydrops (DEH) is a disease caused by secondary endolymphatic hydrops (ELH) after profound hearing loss [1]. In Japan, the criteria proposed by the committee of the Japan Society for Equilibrium Research in 1987 [2] are used for diagnosing DEH. In these criteria, DEH is categorized into two types: ipsilateral and contralateral types. The diagnostic criteria for ipsilateral type of DEH in Japan are as follows: (1) precedent sensorineural hearing loss in one ear; (2) vertigo attack without fluctuating hearing loss in the contralateral ear several years to a decade after the onset of hearing loss; and (3) exclusion of central nervous system lesions. The diagnostic criteria for contralateral type of DEH are as follows: (1) precedent sensorineural hearing loss in one ear; (2) a fluctuating hearing loss in the contralateral ear with or without vertigo attack several years to decade after the onset of hearing loss; and (3) exclusion of central nervous system lesions. In both types, it is thought that secondary ELH induces symptoms of vertigo and/or fluctuating hearing loss, and ELH is thought to be the true cause of DEH.

Recently, 3 T MRI after intratympanic injection of gadodiamide has enabled us to visualize ELH in Meniere’s disease patients [3], and we proposed that 3 T MRI afforded a more accurate and sensitive evaluation technique than did previous methods, such as electrocochleography and glycerol test [4]. Therefore, 3 T MRI was proposed to be a useful diagnostic tool in cases of DEH [5].

The purpose of this study was to assess the diagnostic performance of 3 T MRI after intratympanic injection of gadodiamide for DEH, and assess the relationship between ELH and vestibular function in the patients diagnosed DEH and confirmed by 3 T MRI.

## Materials and methods

### Patients

Nineteen patients clinically diagnosed with DEH based on the Japanese criteria were enrolled in this study. Eleven patients had ipsilateral DEH and 8 patients had contralateral DEH. No patients presented with any concomitant disease, such as meningitis, or autoimmune or systemic disease, and no patients were treated with an immunosuppressant. Oral osmotic diuretics (isosorbide 90 ml/day) were mainly administered during episodes of hearing loss or vertigo. If the degree of hearing loss or vertigo was severe, prednisolone was administered as judged by the attending doctor. In this study, we defined precedent hearing loss as over 70dB (average hearing levels at 250, 500, 1000, and 2000Hz). Data for age, sex, age at onset, DEH type, etiology of precedent hearing loss, side of precedent hearing loss, average hearing levels at 250, 500, 1000, and 2000Hz, presence of vertigo, presence of hydrops (cochlear and vestibular), caloric testing and cVEMP are presented in Table 1. The Ethics Review Committee of Shinshu University School of Medicine approved the protocol of the study and all patients gave their informed consent prior to participation.

**Table 1:** Clinical data for the patients participating in this study.

								Hydrops				Caloric testing		cVEMP	
								Precedent hearing loss ear		Contralateral ear					
Patient no.	Age, Sex	Age at onset**	Type Of DEH	Etiology of precedenthearing loss	Side of Precedent hearing loss	Hearing level (dB)	Vertigo	Cochlea	Vestibule (%*)	Cochlea	Vestibule (%*)	Precedent hearing loss ear	Contralateral ear	Precedent hearing loss ear	Contralateral ear
1	28,F	12	Ipsi	Suspected mumps	R	SO	+	+	+ (68.9)	−	− (31.4)	Hypoflexia	Normal	Absent	Present
2	37,M	34	Ipsi	Childhood onset	L	SO	+	+	+ (66.4)	−	− (26.0)	Normal	Normal	NA	NA
3	45,M	37	Ipsi	Suspected mumps	R	SO	+	−	+ (75.0)	−	− (35.9)	Hypoflexia	Normal	Absent	Present
4	46,F	36	Ipsi	Suspected mumps	L	101.3	+	+	+ (54.6)	−	− (35.7)	Hypoflexia	Normal	Present	Present
5	34,F	33	Ipsi	Suspect of mumps	R	102.5	+	+	+ (53.1)	−	− (28.4)	Hypoflexia	Normal	Absent	Present
6	52,F	51	Ipsi	Sudden deafness	R	70	+	No signal	+ (60.6)	No signal	+ (57.1)	Hypoflexia	Normal	NA	NA
7	47,M	45	Ipsi	Sudden deafness	L	72.5	+	+	+ (75.0)	−	− (31.3)	Hypoflexia	Hypoflexia	Absent	Absent
8	63,F	61	Ipsi	Sudden deafness	R	71.3	+	+	+ (72.0)	−	− (41.7)	Hypoflexia	Normal	Absent	Absent
9	75,M	74	Ipsi	Congenital deafness	L	SO	+	No signal	+ (83.6)	−	− (38.9)	Hypoflexia	Hypoflexia	Absent	Absent
10	38,M	28	Ipsi	Congenital deafness	R	SO	+	+	+ (60.1)	−	− (37.3)	Hypoflexia	Hypoflexia	Absent	Absent
11	44,F	43	Ipsi	Childhood onset	R	71.3	+	+	+ (57.9)	−	− (40.6)	Hypoflexia	Normal	NA	NA
12	73,M	59	Contra	Sudden deafness	L	88.8	−	No signal	Rupture	+	Rupture	Hypoflexia	Hypoflexia	Absent	Absent
13	18,M	15	Contra	Congenital deafness	R	92.5	+	+	− (33.3)	+	+ (50.6)	Hypoflexia	Normal	Absent	Absent
14	73,F	66	Contra	Sudden deafness	R	87.5	+	−	− (38.5)	+	+ (54.3)	Normal	Hypoflexia	NA	NA
15	54,M	53	Contra	Sudden deafness	R	87.5	−	−	+ (51.5)	+	− (41.6)	Normal	Normal	Absent	Absent
16	65,F	64	Contra	Congenital deafness	R	SO	+	−	No signal	+	− (38.4)	Hypoflexia	Normal	Absent	Absent
17	65,M	60	Contra	Childhood onset	R	76.3	−	−	− (31.3)	+	Rupture	NA	NA	NA	NA
18	42,F	41	Contra	Sudden deafness	R	86.3	+	No signal	+ (51.8)	+	− (33.0)	Normal	Normal	Absent	Present
19	55,F	54	Contra	Sudden deafness	R	75	+	+	+ (68.4)	+	− (41.8)	Normal	Normal	Absent	Absent

Ipsi, ipsilateral type; Contra, contralateral type; SO, scale out; +, positive; -, negative; NA, not applicable

* The numbers in parenthesis are the ratio of endolymphatic space to total vestibular space.

** “Age at onset” is the age at which episodes of vertigo in ipsilateral DEH and episodes of fluctuating hearing loss in contralateral DEH commenced.

### MRI

**Fig 1:**
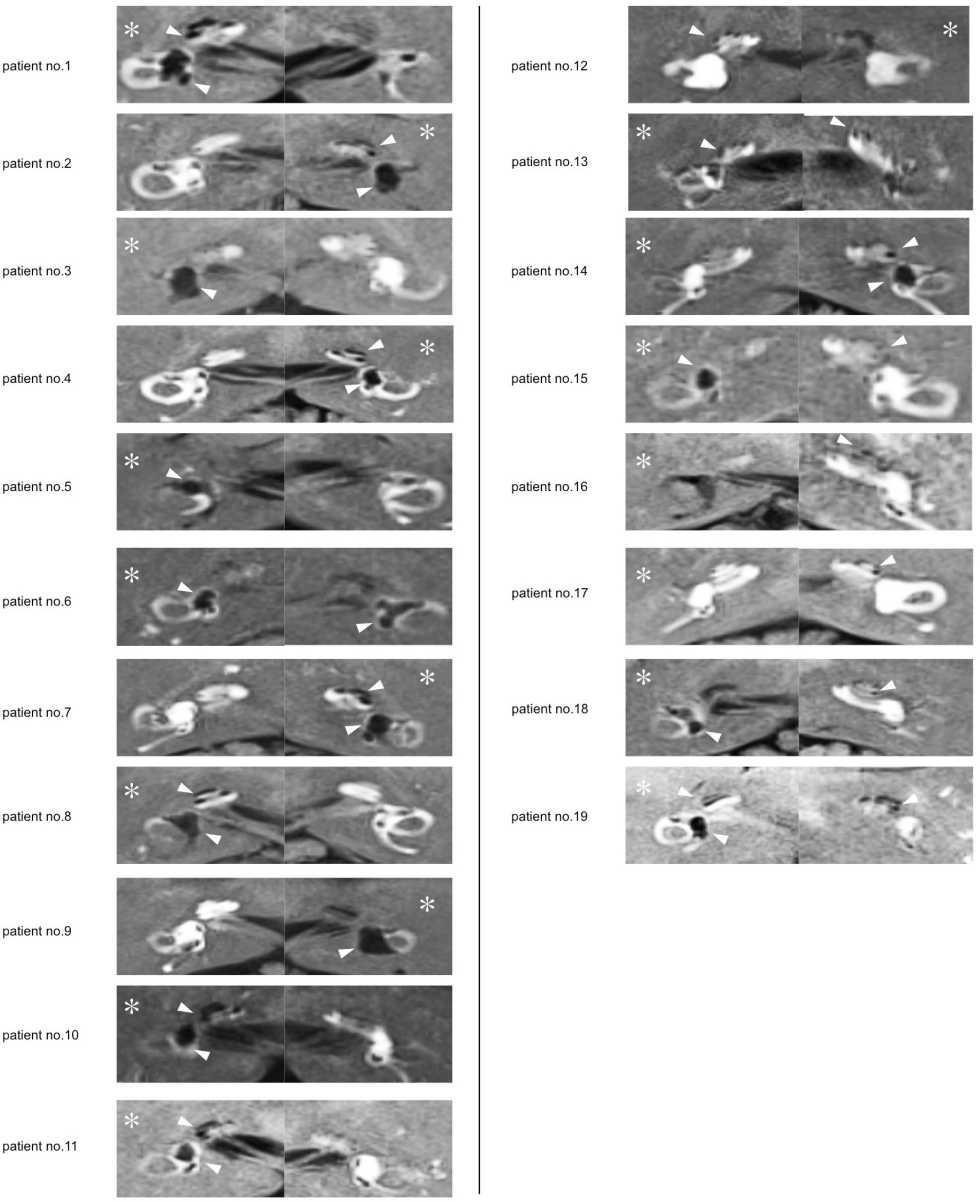
MR images of all patients participating in this study. The perilymph space was filled with gadodiamide and enhanced as a white area. The endolymphatic hydrops (ELH) is detectable as a black area (arrow head). Ipsilateral DEH are shown in the left column and contralateral DEH in the right column. The side of the precedent hearing loss ear is marked with an asterisk.

Gadodiamide (Omniscan, Daiichi Pharmaceutical Co. Ltd, Tokyo, Japan) was diluted eight-fold with saline, and 0.4–0.6 ml of the diluted gadodiamide was administered to the bilateral tympanic cavity by injection through the tympanic membrane using a 23 G needle. The injection was carried out under microscope. The patient then remained in the supine position for 30 minute. After 24 hours, the ELH was evaluated by MRI using a 3.0 T MR scanner (Trio, Siemens, b Erlangen, Germany). The detailed parameters for MR imaging were described elsewhere [4]. Three-dimensional inversion recovery utilizing real reconstruction (3D real IR) imaging was used for the evaluation of ELH. The endolymphatic space of vestibule and total space of vestibule were calculated by sum of each axial slice of the MR image. In this study, when the ratio of the endolymphatic space to total vestibular space exceeded 50%, we judged the patients to have definitive vestibular hydrops. The endolymphatic space of the cochlea was also evaluated from the axial slices of the MR image. When an obvious endolymphatic space in cochlea was determined, we judged the patients to have cochlear hydrops. As shown in Fig 1, cochlear ELH is observed as a nearly rounded extension of the Reissner’s membrane. Three otorhinolaryngologists reviewed the MR images to judge the existence of ELH.

### Vestibular evaluations

#### Caloric testing

Caloric testing involved the measurement of the maximum slow phase velocity (SPV) by cold water irrigation (20°C, 5 ml, 20 s). We defined a maximum SPV value below 20 deg/s as indicative of hypoflexia[6,7].

#### cVEMP

For cVEMP testing, electromyography (EMG) was performed using a pair of surface electrodes mounted on the upper half and sternal head of the sternocleidomastoid (SCM) muscle, respectively. The electrographic signal was recorded using a Neuropack evoked potential recorder (Nihon Kohden Co Ltd, Tokyo, Japan). Clicks lasting for 0.1 ms at 105 dBnHL were presented through a headphone. The stimulation rate was 5 Hz, the bandpass filter intensity was 20 to 2000 Hz, and the analysis time was 50 ms. The responses to 100 stimuli were averaged twice.

## Results

### Ipsilateral DEH

All of the patients (11/11; 100%) with ipsilateral DEH had vestibular ELH in the deafness ear (Table 2). Eight of the 9 patients (8/9; 88.9%) who could be assessed (no cochlear images were obtained for Patient No. 6 and 9) had cochlear ELH in the deafness ear. Patient No. 6 had vestibular ELH in the contralateral ear (1/11; 9.1%) and no patient had cochlear ELH in the contralateral ear (0/11; 0%). As shown in Table 3, caloric testing showed that all patients other than Patient No.2 had vestibular dysfunction in their deafness ears (10/11; 90.1%). In the contralateral ear, caloric testing showed that three patients (No. 7, 9 and 10) had vestibular dysfunction (3/11; 27.3%). cVEMP showed 7 patients had measurable vestibular dysfunction in the deafness ears (7/8; 87.5%); however, half of the patients also had vestibular dysfunction in the contralateral ears (4/8; 50.0%).

**Table 2:** Prevalence of endolymphatic hydrops (ELH) in the cochlea and vestibule of each ear.

Type of DEH	Presence of ELH			
	Precedent hearing loss ear		Contralateral ear	
	cochlea	vestibule	cochlea	vestibule
**Ipsi**	8/9 (88.9%)	11/11 (100%)	0/11 (0%)	1/11 (9.1%)
**Contra**	2/6 (33.3%)	4/7 (57.1%)	8/8 (100%)	4/8 (50%)

Ipsi, ipsilateral type; Contra, contralateral type

**Table 3:** Proportion of vestibular dysfunction in each ear of patients.

Type of DEH	Precedent hearing loss ear		Contralateral ear	
	Caloric testing (hypoflexia)	cVEMP (absent)	Caloric testing (hypoflexia)	cVEMP (absent)
**Ipsi**	10/11 (90.1%)	7/8 (87.5%)	3/11 (27.3%)	4/8 (50%)
**Contra**	3/7 (42.9%)	6/6 (100%)	2/7 (28.6%)	5/6 (83.3%)

Ipsi, ipsilateral type; Contra, contralateral type

### Contralateral DEH

All of the patients with contralateral DEH (8/8; 100%) had cochlear ELH in the better hearing ear (Table 2). Neither the vestibular endolymph region of Patient No.12 nor the left vestibular endolymph region of Patient No.17 was detected. We supposed that rupture of endolymphatic region had occurred and the gadodiamide extend not only to the perilymph but also the endolymph. Therefore, we considered that ELH was present in both vestibules in Patient No.12 and in the left vestibule in Patient No.17. Four of the 8 patients (4/8; 50.0%) with contralateral DEH had vestibular ELH in the better hearing ear. Three of 6 patients (2/6; 42.9%) who could be evaluated (no cochlear image was obtained for Patient No.12 and No.18) had cochlear ELH in the deafness ear. Four of the 7 patients (4/7; 57.1%) who could be evaluated (no vestibular image was obtained for Patient No.16) had vestibular ELH in the deafness ear. Caloric testing showed that two of 7 patients had vestibular dysfunction in the better hearing ear (2/7; 28.6%) and 3 patients had vestibular dysfunction in the deafness ear (3/7; 42.9%). cVEMP showed bilateral vestibular dysfunction in 5 of 6 patients who could be measured (5/6; 83.3%). There were no obvious relationships between ELH and vestibular function.

## Discussion

In this study, we analyzed 19 patients with DEH confirmed by 3 T MRI after intratympanic injection of gadodiamide and herein present the MR images of all patients. ELH was found in all patients (19/19: positive rate is 100%). The true cause of DEH is thought to be secondary ELH. By using 3 T MRI, we could visually show that patients who were clinically diagnosed with DEH actually presented with ELH in their inner ears. Previous reports also indicated that ELH is commonly observed in DEH patients [5,8,9]. Although intravenous methods have also been used to detect ELH [10], our institution basically chose the intratympanic injection method as we believe this method can afford more detailed images than those obtained using the intravenous method. The intratympanic injection method is thought to be safe; in fact, no patient’s audiometric results became worse and no other adverse events were observed (data not shown).

In ipsilateral DEH patients, all of the patients had vestibular ELH in the precedent hearing loss ear. This result is compatible with the disease concept of ipsilateral DEH; precedent hearing loss in one ear causes delayed hydrops in the vestibule of the deafness ear. We investigated the vestibular function of these patients, and most had vestibular dysfunction in their deafness ear as assessed by caloric testing. However, a number of the DEH patients did not have vestibular dysfunction as assessed by caloric testing and some patients had bilateral vestibular dysfunction as assessed by caloric testing and cVEMP. In this study, ipsilateral DEH patients tended to have vestibular dysfunction as assessed by caloric testing of their ELH in the precedent hearing loss ear; however, some of the patients had bilateral vestibular dysfunction as assessed by caloric testing and cVEMP, and we could not find clear a relationship between ELH and vestibular dysfunction.

In contralateral DEH patients, all of the patients had cochlear ELH in the better hearing ear (fluctuating hearing ear). This result is compatible with the disease concept of contralateral DEH; delayed hydrops in the cochlea of better hearing ear induces fluctuating hearing loss. However, there were individual differences in the distribution patterns of ELH in regions other than the cochlea of the better hearing ear (Table 3). Moreover, there was no obvious relationship between ELH and vestibular function, as indicated in the ipsilateral DEH patients. Particularly in the case of contralateral DEH, it is difficult to identify the existence of ELH or which ear, or even which region (cochlea or vestibule or both) has ELH on the basis of vestibular functional testing, such as caloric testing and cVEMP, alone. In a previous report, these forms of vestibular testing provided only limited evidence for the diagnosis of DEH [11]. The direction of the nystagmus is useful for estimation of the affected side; however, we are not always able to observe the nystagmus in patients at the time of a vertigo attack. It is important to determine which ear is responsible for the vertigo for accurate treatment of the disease. We think a more complex pathology is associated with contralateral type DEH than ipsilateral type DEH. ELH distribution was complicated, particularly in the contralateral DEH cases; therefore, 3 T MRI is thought to be useful for accurate evaluation, particularly in cases of contralateral type DEH. The reason why contralateral DEH has two types (with vertigo and without vertigo) is thought to be that some patients have vestibular ELH in either or both ear and some do not have vestibular ELH. DEH might have various etiologies, which may explain differences in the distribution patterns of ELH. Further study is necessary to clarify the detailed pathogenesis of ELH.

We found six patients (1 ipsilateral DEH, 5 contralateral DEH) with bilateral ELH, and believe that such cases should be referred to as bilateral type ELH. For example, Patient No.19 had fluctuating hearing loss in her left ear and also had vertigo. The 3 T MRI indicated no ELH in the left vestibule; however, ELH was identified in the right cochlea and vestibule. Caloric testing showed no vestibular dysfunction, and cVEMP showed dysfunction in both vestibules. Therefore, it is difficult to distinguish which ear had ELH based on vestibular testing. In this case, it is thought that her left cochlear ELH induced the left fluctuating hearing loss and her right vestibular ELH induced the vertigo attacks. Clinically, we diagnosed her symptoms as contralateral DEH; however, her pathology as observed by 3 T MRI should be referred to as “bilateral DEH”. Bilateral DEH is generally considered to occur in patients who have precedent bilateral hearing loss and subsequent episodes of vertigo. However, the definition criteria of bilateral DEH have not been determined clearly. We have experienced some “bilateral DEH” cases in this study. A significant number of cases had “bilateral DEH”, particularly among the contralateral DEH cases, indicating that we should pay careful attention to this pathology when treating DEH. We believe that the diagnostic criteria for DEH will change due to image-based diagnosis using 3 T MRI after intratympanic injection of gadodiamide providing an opportunity for more accurate evaluation.

## Supporting information

S1 Table The ratio of endolymphatic space to total vestibular space. (XLSX)
